# Accuracy of rapid point-of-care serological tests for leprosy diagnosis: a systematic review and meta-analysis

**DOI:** 10.1590/0074-02760220317

**Published:** 2022-04-08

**Authors:** Carmen Phang Romero, Rodolfo Castro, Pedro Emmanuel A do Brasil, Daniella R Pereira, Roberta Olmo Pinheiro, Cristiana M Toscano, Maria Regina Fernandes de Oliveira

**Affiliations:** 1 Fundação Oswaldo Cruz-Fiocruz, Centro de Desenvolvimento Tecnológico em Saúde, Rio de Janeiro, RJ, Brasil; 2 Fundação Oswaldo Cruz-Fiocruz, Escola Nacional de Saúde Pública Sérgio Arouca, Rio de Janeiro, RJ, Brasil; 3 Universidade Federal do Estado do Rio de Janeiro, Rio de Janeiro, RJ, Brasil; 4 Fundação Oswaldo Cruz-Fiocruz, Instituto Nacional de Infectologia Evandro Chagas, Rio de Janeiro, RJ, Brasil; 5 Fundação Oswaldo Cruz-Fiocruz, Brasília, DF, Brasil; 6 Fundação Oswaldo Cruz-Fiocruz, Instituto Oswaldo Cruz, Laboratório de Hanseníase, Rio de Janeiro, RJ, Brasil; 7 Universidade Federal de Goiás, Goiânia, GO, Brasil; 8 Conselho Nacional de Desenvolvimento Científico e Tecnológico, Instituto de Avaliação de Tecnologias em Saúde, Porto Alegre, RS, Brasil; 9 Universidade de Brasília, Brasília, DF, Brasil

**Keywords:** accuracy, diagnosis, leprosy, *Mycobacterium* infections, point-of-care, systematic review

## Abstract

**BACKGROUND:**

Leprosy is a chronic infectious disease, still endemic in many countries that may lead to neurological, ophthalmic, and motor *sequelae* if not treated early. Access to timely diagnosis and multidrug therapy (MDT) remains a crucial element in the World Health Organization’s strategy to eliminate the disease as a public health problem.

**OBJECTIVES:**

This systematic review aims to evaluate the accuracy of rapid point-of-care (POC) tests for diagnosis of leprosy.

**METHODS:**

Searches were carried out in electronic databases (PubMed, EMBASE, CRD, Cochrane Library and LILACS) in April 2021 for patients with suspicion or confirmatory diagnostic of leprosy, classified in multibacillary (MB) or paucibacillary (PB) cases, performing rapid POC serological tests compared to clinical evaluation, smear microscopy and immunohistochemistry analysis. Methodological quality was assessed using the Quality Assessment of Diagnostic Accuracy Studies tool (QUADAS-2). A meta-analysis was undertaken to generate pooled estimates of diagnostic parameters, presenting sensitivity, specificity and diagnostic odds ratio (DOR) values. The review protocol was registered at PROSPERO, CRD # 42014009658.

**FINDINGS:**

From 893 potentially relevant references, 12 articles were included reporting 16 diagnostic tests accuracy studies with 5395 individuals enrolled. Meta-analysis of NDO-LID and PGL-I tests data in MB patients showed sensitivity and specificity [95% confidence interval (CI)] of 0.83 (0.71-0.91), 0.91 (0.72-0.97); and 0.92 (0.86-0.96), 0.93 (0.78-0.98); respectively, with high heterogeneity among the studies.

**MAIN CONCLUSIONS:**

Our results can inform policymakers regarding the possibility of implementing accurate, rapid POC tests for leprosy in public health services, especially within primary health care.

Leprosy is a chronic infectious disease caused mainly by *Mycobacterium leprae*, which is still endemic in many countries in the world. It is characterised by dermato-neurological wounds, presenting with varying clinical manifestations, and may lead to neurological, ophthalmic and motor sequelae if not treated early. Several classifications have been proposed for leprosy. Ridley and Jopling proposed the spectral classification system in 1966, considering bacterial load and cellular immune response. At the two ends of the spectrum, there are the tuberculoid and lepromatous stable forms, and in between with the following unstable intermediate forms: borderline tuberculoid, borderline borderline and borderline lepromatous leprosy.^1^ ^,^ ^2^ ^,^ ^3^ ^,^ ^4^

The disease has a global distribution, except in most European countries, where practically no cases are reported. The number of prevalent global cases at the end of 2019 was 177,175, while the number of new cases detected during 2019 was 202,185.^5^

The countries with the highest leprosy burden are India, Brazil and Indonesia, and they reported more than 10,000 new cases in 2019. The South-East Asia Region accounted for 71.3% of the global leprosy burden. Despite the main target of Global Leprosy Strategy (2016-2020) implemented was to reduce the rate of grade-2 disability (G2D) to < 1 case per million population in two of the most highly endemic countries (Brazil and Indonesia), the number of new cases and cases with G2D has been more or less unchangeable in the past five years.^5^

Access to timely diagnosis and multidrug therapy (MDT) remains a crucial element in the World Health Organization (WHO) strategy to eliminate the disease as a public health problem. The goal is to achieve an incidence of less than 1 case per 10,000 inhabitants; thus, early detection is a priority for disease control.^6^

The diagnosis of leprosy should be suspected when individuals present skin lesion with partial or total loss of thermal, painful and/or tactile sensitivity, with or without thickened nerves. Currently, there is no gold standard test for laboratory confirmation of *leprosy*. *Bacilloscopy* and histopathology studies are complementary exams, and a positive bacilloscopy of an intradermal smear is considered a confirmatory test. However, a negative result does not exclude the diagnosis.^1^ ^,^ ^7^

Clinical diagnosis requires expertise in leprosy and, despite being minimally invasive and of low cost, direct smear microscopy is not always available. Thus, most patients are diagnosed late in the course of the disease when nerve and skin injuries are visible and damage has already occurred.^7^ ^,^ ^8^

Given the barriers to diagnosis and classification of cases, essential to initiate and define treatment, WHO recommended an operational classification based on the count of skin lesions.^9^ Patients with less than five lesions, and five or more, are defined as paucibacillary (PB) and multibacillary (MB), respectively. This classification has low sensitivity and specificity, leading to errors in classification and thus therapeutic choice.^9^ ^,^ ^10^ In Brazil, this classification is based on the identification of skin lesions located in one (PB) or more than one (MB) anatomical region; and/or in one (PB) or more than one (MB) compromised nerve trunk.^11^

The presence of IgM antibody in response to phenolic glycolipid-I (PGL-I), a cell wall component of *M. leprae*, has been proposed to be used in the classification, reducing the errors in clinical diagnosis that may affect the therapeutical choices. In addition, serology is important for selection of household contacts with a higher risk of developing the disease and for case monitoring, as a predictive test for leprosy reactions, for the identification of the risk of relapse.^12^ Since immunity against *M. leprae* is characterised by both humoral and cellular markers, other techniques based on multi-biomarkers evaluation have been developed.^13^ ^,^ ^14^

Point-of-care (POC) or bedside tests, defined as diagnostic tests delivered near or at the place of patient care, are thus critical for timely diagnosis of leprosy patients. When diagnosed early, patients can also be treated promptly, resulting in better clinical outcomes and prognosis and reduced disease dissemination.^15^

ML-Flow methodology was developed in 2003, as an alternative to serology by enzyme-linked immunosorbent assay (ELISA) to assist in classifying patients in PB and MB and in the therapeutic decision in places with difficult access to reference services.^16^ ^,^ ^17^ ^,^ ^18^ Studies using PGL-I have shown that leprosy patients at the lepromatous pole of the spectrum have higher titers of IgM against the antigen (seropositivity: 80-100%), while patients at the tuberculoid pole have immunoglobulin at low levels of detection (seropositivity: 30-60%).^19^

Several studies evaluated not only the diagnostic properties of PGL-1 antigens but also NDO-HAS, a conjugate formed by natural octyl disaccharide bound to human serum albumin, LID-1, the fusion protein product of ml0405 and ml2331 genes, and NDO-LID, a combination of LID-1 and NDO.^20^ ^,^ ^21^ ^,^ ^22^ The RT LID-1 uses a dual-path platform based on the lateral flow technique and the serologic reaction and, it is visible in 10 minutes. Some evidence about its development^21^ ^,^ ^23^ and good performance in its application in the animal model,^22^ as well as about the principle of the technique^24^ was published. However, a recent systematic review and meta-analysis found that LID-1 did not provide any advantage regarding the overall sensitivity estimate compared to PGL-I or ND-O-BSA using the ELISA technique.^25^ ^,^ ^26^

Despite the POC tests developed and made available for use in the last decade, there is a lack of high-level evidence of the accuracy of commercially available POC tests and, particularly, the gap of systematic reviews or meta-analyses studies. Our research question is if rapid POC serological tests are accurate to identify people with leprosy when compared with conventional diagnosis currently used. This work aimed to systematically review the literature on the accuracy of rapid POC serological tests for the diagnosis of leprosy through primary diagnostic studies.

## MATERIALS AND METHODS

*Protocol and guidelines* - We followed the Preferred Reporting Items for Systematic Reviews and Meta-Analyses,^27^ and the review protocol was registered at the International Prospective Register of Systematic Reviews (PROSPERO; CRD # 42014009658). It is available from: https://www.crd.york.ac.uk/prospero/display_record.php?RecordID=9658.

*Information sources and search strategies* - The search for scientific evidence was carried out until April 2021 in five electronic databases: MEDLINE through PubMed, Cochrane Library/Willey, EMBASE, Virtual Health Library and LILACS. The construction of the search strategy used controlled vocabulary terms specific to the databases; “MeSH” terms in MEDLINE, “EMTREE” terms in EMBASE and Cochrane Library bases, and “DeCS” terms in LILACS base.

The search strategy used in MEDLINE was adapted to searching into the other bases. Supplementary data (Table I) presents the strategies for each bibliographic database. To avoid publications certainly not relevant to fit the eligibility criteria of our accuracy systematic review, a searching strategy was translated with no automatic explosion for all terms, and considering specific characteristics of each database.

We also followed the recommendations on how to search the literature for studies evaluating the accuracy of diagnostic tests.^28^ There was no restriction of publication year or language. Additionally, the reference lists of the selected articles were manually scrutinised for the search of primary studies that could have been lost in the electronic search.

*Inclusion and exclusion criteria* - The research question that guided this systematic review was: Are rapid point-of-care serological tests accurate for leprosy detection? The inclusion and exclusion criteria are presented in Table I.

Outcomes of interest were considered when they were reported as such, or when any information in tables or text regarding the number of cases and non-cases with positive or negative test results, considering the index test (e.g., POC test being evaluated) when compared to a reference test (which, on many occasions, where more than one, including various different tests as references for comparison).

**TABLE I:** Eligibility criteria for the selection of studies

	Inclusion	Exclusion
Population	Patients with clinical diagnosis of leprosy Only endemic controls were considered.	Animal model testing. Patients with comorbidities: human immunodeficiency virus (HIV), tuberculosis.
Intervention	Point-of-care (POC) serological tests (defined as the index test).	Laboratory serological tests for research purposes enzyme-linked immunosorbent assay (ELISA), polymerase chain reaction (PCR). Cytological, histological or immuno-histopathological diagnostic studies.
Comparator	Conventional diagnosis: based on clinical signs and symptoms; skin lesion compatible with leprosy and with permanent loss of thermal, painful and/or tactile sensitivity, with or without thickened nerves. A positive smear of an intradermal smear is considered a confirmatory test; however, a negative result does not exclude the diagnosis of leprosy. Ridley and Jopling (R&J) classification for diagnosis and classification	Only classification of cases by operational classification (CO) based on the count of skin lesions, typifying patients with less than five lesions as paucibacillary (PB) and with five or more lesions as multibacillary (MB).
Outcomes	Primary: sensitivity (Se), specificity (Sp), positive likelihood ratio (LR+) and negative likelihood ratio (LR-). Clinical: early detection of leprosy.	No information on the number of patients, positive and negative test results, primary outcomes: sensitivity, specificity; neither information that could allow their calculation.
Study design	Primary studies of diagnostic accuracy, including those with reliable abstracts, tables and additional information that allow extracting data from the study (2x2t able).	Technology development studies. Case report.

*Study selection* - Initially, titles and abstracts were reviewed for inclusion criteria, and duplicates were removed. Full texts of papers meeting the inclusion criteria were retrieved and checked for their eligibility through complete reading.

The process of screening citations and selecting articles was carried out independently by two researchers involved (CPR, RC), and the discrepancies were resolved by consensus. The Rayyan QCRI web application [https://rayyan.qcri.org/welcome^29^] and Mendeley were used to screen and manage the references, respectively; both are open-access software.

*Data extraction* - Data extraction was conducted independently by two reviewers (CPR, RC) using a form in the REDCap platform^30^ specifically configured for this systematic review, and any discrepancies were resolved by a third reviewer (CT). Data extracted from each study included authors, year of publication, country, funding source, study design, index diagnostic test details (manufacturer, type of test, laboratory method used, etc.), study setting and period, baseline information on study population, case definition, leprosy diagnostic criteria and exclusion criteria reported, standard reference test considered, and measure of outcome reported. The extracted data were used to build 2x2 tables for each study, the same ones that were used to recalculate accuracy values and to verify the data presented in each publication.

*Methodological quality assessment* - The quality of the evidence was assessed using the Quality Assessment of Diagnostic Accuracy Studies tool (QUADAS-2).^31^ QUADAS-2 is structured around four domains of potential sources of bias in primary diagnostic studies: patient selection; index test; reference standard; and flow and time. Each domain is assessed in terms of risk of bias, and the first three domains are also assessed in terms of concerns regarding applicability. Concerns about applicability are rated as “low,” “high,” or “unclear” and the authors used the “unclear” category when insufficient data were reported to permit judgment. The instrument also provide signaling questions to help judge risk of bias (more details could find in the QUADAS-2 reference).^31^ This step was also performed independently by the reviewers (CPR and DP) and any discrepancies resolved by a third reviewer (MRF).

*Data analysis* - Sensitivity and specificity values were recalculated for each study included in our review, considering criteria standardised by the investigators after consulting with leprosy experts. Gold standard definition of case and non-case was considered the patients diagnosed by the clinical criteria and WHO’s operational classification^9^ and healthy subjects, respectively. Accuracy measures were estimated for MB and PB patients separately and to estimate the percentage of each one the denominator used was the total sick population (MB+PB). When recalculating accuracy estimates, we considered only endemic controls, even when other types of controls were reported by the authors. Household contacts with a positive serology have a higher chance to develop the disease.^32^ These findings suggest that positive serology in some contacts might be a marker of subclinical infection. We don’t have a marker that discriminates exposure from subclinical infection.

The process of data synthesis comprises qualitative analysis to identify clinical or methodological heterogeneity and quantitative assessment through statistical tests of χ² (Cochran Q) and I² for more objective assessments of heterogeneity.

Data synthesis was performed using mixed-effects logistic regression models with maximum likelihood estimation based on the adaptive Gaussian square using “xtmelogit” in the MIDAS package (Available from: https://ideas.repec.org/c/boc/bocode/s456880.html).

From the statistical analysis, the calculation of the summary estimate of diagnostic accuracy was obtained for the set of studies. However, due to the clinical heterogeneity observed, statistical analyses were performed by subgroups, by test performance in MB and PB patients, for both NDO-LID and PGL-I tests.

Meta-analysis was performed using a two-level model, within the bivariate mixed-effects binary regression modeling framework, comprising exact binomial approach for within-study variability, and logit transforms of sensitivity and specificity for between-study variability. The results of the meta-analysis are presented in graphs (forest plots) with a 95% confidence interval (CI) for sensitivity and specificity. Positive and negative likelihood ratios and diagnostic odds ratio (DOR) were reported for each type of test. The summary receiver operating characteristic (SROC) curve presents a summarised operational point and 95% confidence and prediction contours.

Two forest plots are presented side by side: one for sensitivity and the other for specificity. Moreover, the number of true positives, false positives, true negatives and false negatives are also reported, where they were appropriate.

Statistical analyses were performed using STATA statistical software (StataCorp. 2017. Stata Statistical Software: Release 15. College Station, TX: StataCorp LLC).

*Ethical approval* - Not required.

## RESULTS

**Fig. 1:**
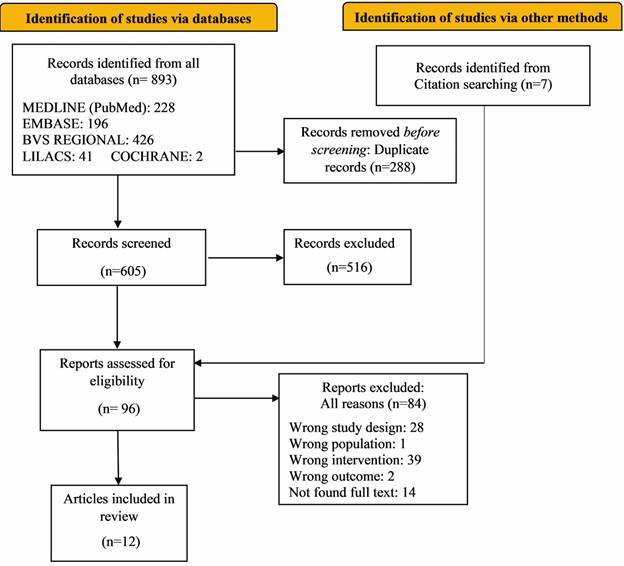
PRISMA 2020 - Flow chart of study selection process.

From structured searches, 893 potentially relevant references were found; after removing duplicates and screening titles and abstracts, 96 full-text references were analysed for eligibility criteria, 74 articles were further excluded and nine were not available. Finally, 12 articles were included in the review (Fig. 1).

A total of 5395 individuals were enrolled in the studies, and 16 diagnostic tests accuracy assessments were reported by 12 studies included in this review.

One study^33^ assessed the same index test in two populations (Brazil, Nepal) with different characteristics; the other two studies^34^ ^,^ ^35^ assessed the same index test from two different manufacturers, then all were analysed separately and assigned with code “a” and “b”.

The summary of 16 included diagnostic tests characteristics is presented in Table II. The studies were carried out in 10 countries: Brazil, Bangladesh, Colombia, China, Ethiopia, Ghana, India, Indonesia, Nepal and Philippines, 31.25% of them as multicentre studies, most of them undertook in hospitals, research centres or institutes (87.5%) and covering ten different manufacturers of the index test.

**TABLE II:** Summary of characteristics from the 16 tests assessed by 12 studies

Characteristics	Number of studies n (%)
Manufacturer	
Orange Life (Rio de Janeiro) Brazil	5 (31.25)
CTK Biotech (San Diego CA)	2 (12.5)
KIT (Biomedical Research) The Netherlands	2 (12.5)
Standard Diagnostics (Yongin South Korea)	1 (6.25)
InBios International Inc. Seattle WA USA	1 (6.25)
Yonsei University and Standard Diagnostics Inc (Suweon Republic of Korea)	1 (6.25)
OmegaTeknika Limited (Dublin Ireland) and Royal Tropical Institute (KIT Amsterdam Netherlands)	1 (6.25)
Amrad ICT Diagnostics (Brookvale) NSW	1 (6.25)
UPCON; Labrox, Finland	1 (6.25)
Instituto de Patologia Tropical e Saúde Pública (IPTSP), Universidade Federal de Goiás	1 (6.25)
Antigen - Index test	
Phenolic glycolipid-I (PGL-I)	5 (31.25)
NDO-LID conjugate	9 (56.25)
PGL-I + (IP-10, CCL4 and CRP)	1 (6.25)
35-kD test	1 (6.25)
Publication year	
2019	2 (12.5)
2018	2 (12.5)
2017	2 (12.5)
2016	2 (12.5)
2014	3 (18.75)
2012	2 (12.5)
2008	1 (6.25)
2003	1 (6.25)
1999	1 (6.25)
Multicentric investigation	
Yes	6 (37.5)
No	10 (62.5)
Study setting	
Primary care	2 (12.5)
Secondary care	7 (43.75)
Tertiary care	7 (43.75)
Country were study conducted^*^	
Brazil^*^	8 (50.0)
Bangladesh^*^	1 (6.25)
Colômbia	1 (6.25)
China^*^	1 (6.25)
Ethiopia^*^	1 (6.25)
Ghana^*^	1 (6.25)
India	1 (6.25)
Indonesia^*^	1 (6.25)
Nepal^*^	3 (18.75)
Philippines^*^	5 (31.25)

*: multicentric studies, total sum not equal to 16.

Study sample sizes varied from 140 to 780 patients per study. The ages of the recruited participants ranged from 10 to 81 years old, but we could not estimate the mean age of participants because that information was missing in some studies. The proportion of male individuals participating was 60.5%, reported in 10 out of the 16 studies included. Regarding classification of cases, MB patients were more frequent (65%) than PB cases.

The characteristics of the index and standard reference tests are shown in Table III. Very few losses were reported in the studies: only one study^36^ reported loss of 53 (12.2%) participants because they refused blood collection. In all studies included, the biological sample tested by the index test was either serum, whole blood or both.

**TABLE III:** Summary of tests characteristics from the 16 included studies

Study, year	Cohort	Index test	Biological sample	Standard reference	N° participants enrolled	N° participants included in analysis^*^	% MB
Leturiondo AL, 2019	Brazil	NDO-LID conjugate	Serum	CO + Bacilloscopy + R&J	780	701	70.76%
Leturiondo AL, 2019	Brazil	Phenolic glycolipid-I (PGL-I)	Serum	CO + Bacilloscopy + R&J	701	70.76%
Góis RS, 2018	Brazil	NDO-LID conjugate	Whole blood	CO + Bacilloscopy	140	125	31.43%
Hooij AV, 2018	Brazil, China, Ethiopia	PGL-I + (IP-10, CCL4, CRP)	Whole blood	CO + R&J	715	467	63.00%
Frade MAC, 2017	Brazil	NDO-LID + Smart Reader	Whole blood	CO + R&J	434	288	88.37%
Hooij AV, 2017	Philippines, Bangladesh	NDO-LID conjugate	Serum	CO + R&J + BI	434	434	74.82%
Duthie MS, 2016^1^	Philippines	NDO-LID conjugate^1^	Whole blood	CO + Bacilloscopy + R&J	635	102	NA
Duthie MS, 2016^2^	Philippines	NDO-LID conjugate^2^	Whole blood	CO + Bacilloscopy + R&J	102	NA
Duthie MS, 2014^1^	Philippines	NDO-LID conjugate^1^	Serum	CO + Bacilloscopy + R&J	384	333	77.04%
Duthie MS, 2014^3^	Philippines	NDO-LID conjugate^3^	Serum	CO + Bacilloscopy + R&J	333	77.04%
Duthie MS, 2014	Colombia, Philippines	NDO-LID conjugate	Whole blood	CO + Bacilloscopy + R&J	388	299	74.49%
Stefani M, 2012^4^	Brazil, Nepal	Phenolic glycolipid-I (PGL-I)^4^	Both (Serum+WB)	CO + Bacilloscopy + R&J	363	239	50.00%
Stefani M, 2012^5^	Brazil, Nepal	Phenolic glycolipid-I (PGL-I)^5^	Both (Serum+WB)	CO + Bacilloscopy + R&J	363	139	49.64%
Parkash O, 2008	India	Phenolic glycolipid-I (PGL-I)	Whole blood	CO + Bacilloscopy	209	172	17.01%
Bührer-Sékula S, 2003	Brazil, Indonesia, Philippines, Ghana	Phenolic glycolipid-I (PGL-I)	Both (Serum+WB)	CO	739	433	57.29%
Roche P, 1999	Nepal	35-kD test card^*^	Serum	R&J	174	97	55.56%

CO: clinical observation; R&J: Ridley & Joppling classification; PB: paucibacillary; MB: multibacillary; NA: not available. (*) Antigenic protein point-of-care (POC) tests. ^1^Orange Life (Rio de Janeiro/Brazil); ^2^CTK Biotech (San Diego CA); ^3^Standard Diagnostics (Yongin South Korea); ^4^OmegaTeknika Limited (Dublin Ireland) and Royal Tropical Institute (KIT Amsterdam Netherlands); ^5^Yonsei University and Standard Diagnostics Inc (Suweon Republic of Korea).

The reference test varied among the studies. In general, the R&J classification, clinical observation and smear microscopy were used in the studies, reporting the results separately most studies (9/16) used the three diagnostic criteria together (Table III).

List of excluded articles and reasons for exclusion are described in Supplementary data (Table II).

Among the 16 assessed diagnostic tests by 12 studies, nine were NDO-LID conjugate,^26^ ^,^ ^36^ ^-^ ^41^ five Phenolic glycolipid-I (PGL-I),^33^ ^,^ ^41^ ^,^ ^42^ ^,^ ^43^ one study was PGL-I + [IP-10, CCL4 and C-reactive protein (CRP)],^44^ a combination of serological and additional cellular immune-markers test named Up-Converting Phosphor (UCP) Lateral Flow Assays (LFAs) and another one evaluated the 35-kD antigenic protein POC tests.^45^ From the 9 NDO-LID conjugate tests, two^35^ only presented overall measures of test accuracy. Supplementary data (Table III) shows the accuracy parameters recalculated for the POC tests evaluated by the studies included.

**Fig. 2:**
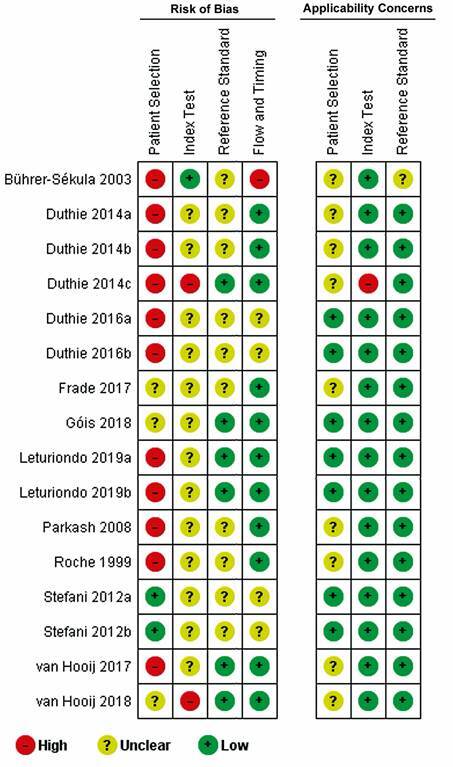
methodological quality summary: review authors’ judgements about each domain for each included study.

*Methodological quality assessment* - Most studies presented moderate quality based on QUADAS-2, by the fact that the risk of bias was difficult to assess due to a high percentage of domains qualified as “unclear”, i.e., when insufficient data were reported to permit judgment (Fig. 2). Nevertheless, risk of bias for the domain patient’s selection was identified in 11 out of the 16 studies. The domains assessing index and reference tests scored “unclear”. Two studies were assessed with a high risk of bias for at least two domains^39^ ^,^ ^46^ (Fig. 2). Applicability concerns were low for both index and reference tests and mostly unclear for patient’s selection. The review authors’ judgements about each domain presented as percentages across included studies are in Supplementary data (Fig. 1).

*Study heterogeneity* - From a methodological point of view,^47^ the sources of heterogeneity among the studies are many, such as, differences in patient and control characteristics; recruitment process with no random or consecutive patient inclusion; not being able to avoid the case-control design; among others. Differences in patient selection and use of controls, were the most frequent source of high risk of bias detected by QUADAS-2.

The leprosy patients cohort from Frade et al.^36^ were known contacts of patients or non-contacts recruited on a mobile clinic (stationed at the main bus terminal in Brasília, Brazil); in Duthie et al.,^35^ volunteers of both sexes and a range of ages were recruited at the Cebu Skin Clinic; in Bührer-Sékula et al.,^43^ newly diagnosed and treated leprosy patients from three areas of high leprosy endemicity (Manaus in Brazil, South Sulawesi in Indonesia, and Cebu in Philippines) were selected.

**Fig. 3:**
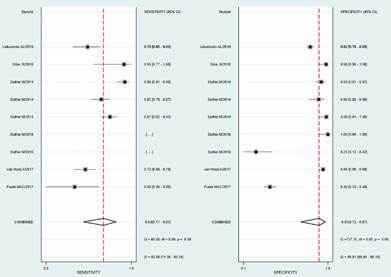
meta-analysis results of NDO-LID tests in multibacillary (MB) cases. Forest plot showing sensitivity and specificity of NDO-LID tests for MB cases.

*Performance of tests* - Meta-analysis of NDO-LID tests in MB patients (Fig. 3) showed a sensitivity of 0.83 (0.71-0.91 95% CI) and specificity of 0.91 (0.72-0.97 95% CI), Positive Likelihood Ratio 9.1 [2.5-33.4], Negative Likelihood Ratio 0.19 [0.10-0.36], DOR 49 [7-316] and heterogeneity among studies remained high either for sensitivity or specificity estimation, I^2^ = 83.86 (74.38-93.34); I^2^ = 98.91 (98.64-99.19), respectively. The SROC curve shows an area under ROC of 0.92 (0.89-0.94) [Supplementary data (Fig. 2)].

**Fig. 4:**
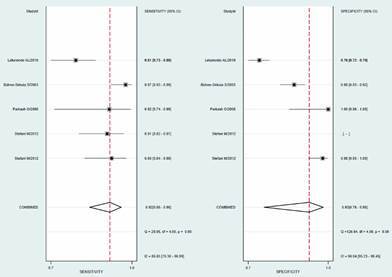
meta-analysis results of PGL-I tests in in multibacillary (MB) cases. Forest plot showing sensitivity and specificity of PGL-I tests for MB cases.

Meta-analysis of Phenolic glycolipid-I (PGL-I) test in MB patients (Fig. 4) showed a sensitivity of 0.92 (0.86-0.96 95% CI) and specificity of 0.93 (0.78-0.98 95% CI), Positive Likelihood Ratio 13.9 [3.7-52.0], Negative Likelihood Ratio 0.08 [0.04-0.16], DOR 166 [30-920] and the degree of heterogeneity was high for the sensitivity I^2^ = 86.65 (76.30-96.99) and specificity estimates I^2^ = 96.84 (95.23-98.45). The SROC curve in an area under ROC of 0.97 (0.95-0.98) [Supplementary data (Fig. 3)].

Estimates for PB patients showed sensitivity of 0.38 (0.14-0.70 95% CI) and specificity of 0.91 (0.72-0.98 95% CI) for NDO-LID tests and, sensitivity of 0.36 (0.32-0.41 95% CI) and specificity of 0.94 (0.75-0.99 95% CI) for PGL-I tests [Supplementary data (Figs 4-7)].

The new test UPC-LFAs showed a sensitivity of 0.85 and specificity of 0.75. Only one study performing the 35-kD antigenic protein tests^45^ showed a sensitivity of 0.68 and specificity of 0.90. None of these tests was included in the meta-analysis.

## DISCUSSION

We were able to estimate the diagnostic accuracy of rapid tests that can be implemented in public health systems to improve early detection and prompt treatment for leprosy patients. Both NDO-LID and PGL-I tests presented reasonable accuracy for MB cases. Even though PGL-I showed slightly better sensitivity, specificity and DOR, results must be interpreted with care since few studies were analysed. Results are not applicable for PB leprosy cases because in these patients, the antibody response is generally absent. However, biomarkers of the cellular immune response are observed in PB cases, which open the perspective for developing strategies that combine both cellular and humoral immune responses evaluation.^44^ ^,^ ^48^

Technologies developed for the diagnosis of leprosy are guided by the patient’s cellular and humoral immune response. Even though all accuracy measures were estimated for MB and PB patients, the natural history of the disease shows that humoral immunity is set in MB. At the same time, PB mainly presents cellular immunity, which is not easily assessed through either POC immunochromatographic or immunoassays.^49^ Identifying a host biomarker signature to support diagnosis has made a significant advance, with high titters of anti-PGL-1 IgM, IP-10/CXCL-10 and CRP being associated with leprosy.^50^

Recently, a multi-biomarker test (MBT) evaluating αPGL-I IgM, IP-10, CRP, S100A12, and ApoA1 was successfully used to discriminate patients with MB and PB leprosy from control individuals in high and low leprosy endemic areas.^13^ However, these data should be replicated in larger and different cohorts and this study had a limitation in that it did not exclude treated patients and it is known that treatment can affect the titters of anti-PGL-1 in sera from leprosy patients. However, previous studies from the same group evaluated immunodiagnostic tests in three different cohorts from Brazil, China and Ethiopia. They evaluated the levels of anti-PGL-1 IgM antibody as well as IP-10, CCL4 and CRP in blood collected from leprosy patients and observed that combined detection of these biomarkers significantly improved the diagnostic potential, particularly for PB leprosy in all studied regions. The combination of cellular and humoral markers increased the test sensitivity from 0.50 to 0.54, resulting in an overall sensitivity for BT/TT leprosy of 0.80 (China), 0.71 (Brazil) and 0.75 (Ethiopia).^44^

Despite the fact that Ridley and Jopling classification^4^ allowed a better understanding of the spectrum of the clinical forms of leprosy, it needs the support of pathological anatomy for its application; its use is intended for specialised centres and research work.^51^ In addition, clinical diagnosis requires well-trained clinicians, and direct smear microscopy is not always available, although it is a low-invasive and low-cost method. In the absence of a laboratory diagnosis and a golden standard test, an individual with cutaneous lesions or symptoms suggestive of nerve damage, with loss of thermal, painful and/or tactile sensitivity, is considered a “suspected case”.^5^ ^,^ ^8^

In practice, clinical criteria are used to classify and decide the appropriate treatment regimen for leprosy patients, particularly considering the non-availability or unreliability of some services to perform the smear exam. The operational classification defines PB and MB patients based on the skin lesions count.^7^ However, it has low sensitivity and specificity, leading to errors in classification and, consequently, the choice of the therapeutic protocol.

One study found^12^ that MB patients who received MDT, only 62.9% had positive serology. Of these, 55.9% would have up to five skin lesions, and therefore should be classified as PB. It is critical to avoid that MB patients be treated with the regimen for the PB form of leprosy, considering that the therapeutic regimen for MB is 12 doses, while for PB patients, it is six doses.

A LID-1 test performed by using ELISA technique points to sensitivity of 81.6% and specificity of 97% in overall patients (MB, PB) when compared to the bacillary index and skin biopsy.^52^ Meanwhile, there is some evidence about the accuracy of based-laboratory tests;^53^ performance assessment of POC tests is still scarce.

In this review nine studies performing NDO-LID conjugate were assessed and demonstrated good SE 0.83 (0.71-0.91 95% CI) and SP 0.91 (0.72-0.97 95% CI), and five studies evaluating Phenolic glycolipid-I (PGL-I) showed better SE 0.92 (0.86-0.96 95% CI) and SP 0.93 (0.78-0.98 95% CI), respectively.

The application of QUADAS instrument^31^ as a tool to assess risk of bias in comparative diagnostic accuracy studies reported a moderate methodological quality of the studies. They scored “unclear” in the domains related to the index and reference tests mainly due to the scarce details of the reports. Patient selection bias was identified on 11/16 studies, in order to prevent this kind of potential bias is recommended an enrollment of a consecutive or random sample of eligible patients with suspected disease. However, applicability concerns were low for both index and reference tests and mostly unclear for patient selection.

Some limitations must be pointed out, such as the small number of studies assessing RT to determine its accuracy, avoiding better quantification of accuracy measures through meta-analyses. Studies selected presented diversity of participants (clinical form of the disease, baseline characteristics and different groups of cases and controls) that could influence the overall measures. The quality of report was poor at most studies due to incomplete characteristics details of the population. We have also observed the inclusion of inappropriate types of controls, as non-endemic controls, which were individuals not at risk of having the disease, the absence of clear description of the patient selection process or an incomplete analysis of cases and control groups. Regarding patient characteristics, studies should be better evaluated by the QUADAS-2, which is the appropriate form of risk of bias assessment. The case-control study design should be avoided, and the patients and controls should be recruited from the same population, including individuals that are randomly or consecutively selected from a unique population of people at risk of the disease. Tests should be assessed in the same circumstances of their future applications in the public health setting. If in the public health setting it is not reasonable to apply the diagnostic tests for non-endemic controls without any characteristics of propensity of getting the disease, these types of patients should not be included in primary accuracy studies. The use of two populations, one of cases that are individuals with high propensity of being truly positive, and another population of controls that are individuals with high propensity of being truly negative, contributed to the high risk of bias observed in the studies included.

Methodological heterogeneity due to selection, design and comparison of the clinical characteristics of the patients may cause potential bias; inclusion criteria varied among the studies and there were no consolidated standards regarding the clinical diagnostic, which may lead to different outcomes. We also need to point out that classical heterogeneity measures such as χ² and I² have limitations for accuracy systematic reviews; however, in our review heterogeneity, it can be clearly detected in the forest plots presented. The statistical estimates of heterogeneity among studies, although high, are of little practical applicability for conclusions regarding variability among studies, since heterogeneity may reflect variability introduced by changes in diagnostic threshold among the studies. The studies could have used different thresholds to define positive and negative test results that cannot be objectively measured.

Previous studies have demonstrated that contacts of leprosy patients with positive serology anti-PGL-1 have a higher chance to develop the disease.^32^ ^,^ ^54^ However, positive serology may be associated with both exposure or subclinical infection. In the present study, we decided to exclude household contacts in order to reduce the heterogeneity in the analysis. Our future studies will evaluate the impact of positive serology on early diagnosis of disease in the contacts which could contribute for the definition of the target population of chemoprophylactic strategies.

Although the absence of contacts in the present study, our results can inform policymakers regarding the possibility of implementing rapid tests (RT) for leprosy in public health services, especially within primary health care. MB patients could be promptly screened using RT strategies, allowing early diagnostic and treatment delivery. These strategies can avoid severe lesions and associated disabilities. Rehabilitation to reduce patient suffering and costs could also be saved.

The results of this review are not conclusive regarding the accuracy of the tests summarised in the meta-analysis; they showed better performance of the PGL-I tests compared to the NDO-LID tests both in sensitivity and specificity parameters but it was also found high degree of heterogeneity among the studies for both tests. This high heterogeneity may reveal the complexity of leprosy disease and also the absence of a gold standard laboratorial test. Further studies must be designed following the guidelines for diagnostic test accuracy studies in order to produce better quality evidence based on standard criteria in a defined clinical context. There is also need to assess the cost-effectiveness of leprosy RT from a public health perspective.
