# Supplementary material for: Accuracy of rapid point-of-care serological tests for leprosy diagnosis: a systematic review and meta-analysis
Source: Mem Inst Oswaldo Cruz. 2022 Apr 8;117:e220317. doi: 10.1590/0074-02760220317 (PMC9000963; doi:10.1590/0074-02760220317)
Supplement: Supplementary file 1 [file 1678-8060-mioc-117-e220317-s.pdf]

TABLE I
Search strategies in bibliographic databases

<table>
<tr><th>DB</th><th colspan="3">Search strategy</th><th>N</th><th>Date</th></tr>
<tr><td>PUBMED</td><td colspan="3">((((((((“Leprosy”[Mesh] OR “Leprosies” OR “Hansen Disease” OR “Disease, Hansen” OR “Hansen’s Disease” OR “Disease, Hansen’s” OR “Hansens Disease”)) AND (“Early Diagnosis”[Mesh] OR “Diagnosis, Early” OR “Early Detection of Disease” OR “Disease Early Detection” OR “Point-of-Care Testing”[Mesh] OR “Point-of-Care Testings” OR “Testing, Point-of-Care” OR “Testings, Point-of-Care” OR “Point of Care Testing” OR “Bedside Testing” OR “Bedside Testings” OR “Testing, Bedside” OR “Rapid test” OR “Screening” OR “Serologic Tests”[Mesh] OR “Serological Tests” OR “Serological Test” OR “Test, Serological” OR “Tests, Serological” OR “Tests, Serologic” OR “Serologic Test” OR “Test, Serologic” OR “Serodiagnosis” OR “Serodiagnoses”))) AND (“Sensitivity and Specificity”[Mesh] OR “Specificity and Sensitivity” OR “Sensitivity” OR “Specificity”))) NOT Tuberculosis) NOT Leishmaniasis) NOT Diabetes</td><td>228</td><td>04/19/21</td></tr>
<tr><td>EMBASE</td><td colspan="3">(‘leprosy’/mj OR ‘mycobacterium leprae infection’ OR ‘hansen disease’ OR ‘hanseniasis’ OR ‘lepra’ OR ‘leprology’ OR ‘leprosis’ OR ‘leprosy’) NOT (‘tuberculosis’/exp OR ‘mycobacterium tuberculosis infection’ OR ‘active tuberculosis’ OR ‘chronic tuberculosis’ OR ‘minimal tuberculosis’ OR ‘minimum tuberculosis’ OR ‘tuberculosis’ OR ‘tuberculosis, cardiovascular’ OR ‘tuberculosis, endocrine’ OR ‘tuberculous infection’ OR ‘tuberculous lesion’) NOT ‘leishmaniasis’/exp AND (‘diagnostic test’/mj OR ‘diagnostic test’ OR ‘diagnostic tests, routine’ OR ‘test, diagnostic’ OR ‘early diagnosis’/mj OR ‘diagnosis, early’ OR ‘early diagnosis’ OR ‘point of care testing’/mj OR ‘bedside testing’ OR ‘point of care testing’ OR ‘point-of-care testing’ OR ‘serology’/mj OR ‘serologic test’ OR ‘serologic tests’ OR ‘serological test’ OR ‘serology’) AND (‘sensitivity and specificity’/mj OR ‘sensitivity and specificity’ OR ‘specificity and sensitivity’ OR ‘accuracy’/mj OR ‘accuracy’ OR ‘precision’)</td><td>196</td><td>04/19/21</td></tr>
<tr><td>BVS</td><td colspan="3">(tw:((leprosy) OR (lepra) OR (hanseníase) OR (doença de hansen) OR (mycobacterium leprae) OR (bacilo de hansen) OR (bacilo da hanseníase))) AND (tw:((diagnosis) OR (diagnóstico) OR (triagem) OR (rastreamento) OR (uso diagnóstico) OR (serologic tests) OR (pruebas serológicas) OR (testes sorológicos) OR (testagem sorológica) OR (diagnóstico sorológico) OR (sorodiagnóstico) OR (early diagnosis) OR (diagnóstico precoz) OR (diagnóstico precoce) OR (point-of-care testing) OR (pruebas en el punto de atención) OR (testes imediatos) OR (testes junto ao leito) OR (testes à beira do leito) OR (diagnóstico junto ao leito) OR (diagnóstico à beira do leito) OR (diagnósticos de cabeceira) OR (diagnósticos junto ao leito) OR (diagnósticos à beira do leito) OR (exames de cabeceira) OR (teste junto ao leito) OR (teste à beira do leito) OR (testes de cabeceira) OR (análises clínicas ambulatoriais) OR (análises clínicas no ambulatório) OR (análises diagnósticas no consultório) OR (análises do paciente no consultório) OR (diagnóstico no local de atendimento) OR (diagnósticos ambulatoriais) OR (diagnósticos no ambulatório) OR (diagnósticos no local de atendimento) OR (exames imediatos) OR (teste point-of-care) OR (teste no consultório) OR (teste no local de atendimento) OR (testes diagnósticos no consultório) OR (testes no consultório) OR (testes no local da intervenção) OR (testes no local de atendimento))) AND (tw:((sensitivity AND specificity) OR (sensibilidad y especificidad) OR (sensibilidade e especificidade) OR (especificidade) OR (verdadeiros positivos) OR (verdadeiros negativos))) AND NOT (tw:(tuberculosis)) AND NOT (tw:(leishmania)) AND NOT (tw:(diabetes))</td><td>426</td><td>04/19/21</td></tr>
<tr><td>LILACS</td><td colspan="3">Search (in Spanish): (lepra OR hansen) AND (diagnóstico OR test OR serolog OR sorolog) AND (sensibilidad OR especificidad) AND (db:(“LILACS”))</td><td>41</td><td>04/19/21</td></tr>
<tr><td rowspan="4">COCHRANE</td><td>#1</td><td>MeSH descriptor: [Leprosy] explode all trees</td><td>293</td><td rowspan="4">2</td><td rowspan="4">04/19/21</td></tr>
<tr><td>#2</td><td>MeSH descriptor: [Immunologic Tests] explode all trees</td><td>5204</td></tr>
<tr><td>#3</td><td>MeSH descriptor: [Sensitivity and Specificity] explode all trees</td><td>15503</td></tr>
<tr><td>#4</td><td>#1 and #2 and #3</td><td>2</td></tr>
</table>

CC BY

TABLE II

List of excluded studies and reasons of exclusion

Ref	Tittle	Year	Journal	Author	Exclusion reasons
1	[Sensitivity and specificity of fluorescent leprosy antibody absorption (FLA-ABS) test for detecting subclinical infection by *Mycobacterium leprae*].	1984	Zhonghua Yi Xue Za Zhi	Ji et al.	Not POC. Not reliable diagnostic test for early leprosy.
2	[The detection of IgM antibodies to phenolglycolipid I for serodiagnosis of Hansen's disease and monitoring the contact population in Polynesia. Five year evaluation].	1990	Bulletin de la Societe de pathologie exotique	Chanteau et al.	Not POC. Laboratory-based
3	A study on performance of two serological assays for diagnosis of leprosy patients.	1995	Lepr Rev	Parkash et al.	Not accuracy study (Concordance between testes)
4	A study on the reproducibility of two serological assays for detection of *Mycobacterium leprae* infection [6]	2001	International Journal of Leprosy and Other Mycobacterial Diseases	Parkash	Not accuracy study. Intra-assay and inter-assay variability
5	Anti-PGL-1 Positivity as a Risk Marker for the Development of Leprosy among Contacts of Leprosy Cases: Systematic Review and Meta-analysis	2016	PLoS Neglected Tropical Diseases	Penna et al.	SR not primary data
6	Application of *Mycobacterium leprae*-specific cellular and serological tests for the differential diagnosis of leprosy from confounding dermatoses	2016	Diagnostic Microbiology and Infectious Disease	Freitas et al.	Not POC. Laboratory-based
7	Association of mycobacterial-specific and *Mycobacterium leprae* specific antibody levels with clinical activity in tuberculoid leprosy: a comparative study of three serological enzyme-immunoassays.	1991	Leprosy review	Chaturvedi et al.	Not accuracy study (Concordance between testes)
8	Comparative assessment of the leprosy antibody absorption test, *Mycobacterium leprae* extract enzyme-linked immunosorbent assay, and gelatin particle agglutination test for serodiagnosis of lepromatous leprosy	1993	J Clin Microbiol	Escobar-Gutierrez et al.	Not POC. Not accuracy study (Concordance between testes)
9	Comparing the sensitivity of auramine-rhodamine fluorescence to polymerase chain reaction in the detection of *Mycobacterium leprae* in Fite-negative tissue sections	2017	Journal of the American Academy of Dermatology	Elston et al.	Publication type - research letter
10	Comparison between anti-PGL-I serology and Mitsuda reaction: clinical reading, microscopic findings and immunohistochemical analysis.	2003	Leprosy review	Maeda et al.	Different intervention - Lepromin test
11	Comparison between microsatellites and Ml MntH gene as targets to identify *Mycobacterium leprae* by PCR in leprosy.	2011	An Bras Dermatol	Cruz et al.	Not POC. Laboratory-based
12	Comparison of three immunological tests for leprosy diagnosis and detection of subclinical infection	2011	Leprosy Review	Lobato et al.	Not accuracy study (Concordance between testes)
13	Comparison of two different PCR amplification products (the 18-kDa protein gene vs. RLEP repetitive sequence) in the diagnosis of *Mycobacterium leprae*	2003	Clinical and Experimental Dermatology	Kang et al.	Not POC. Laboratory-based
14	Detection and quantification of *Mycobacterium leprae* in tissue samples by real-time PCR	2004	Med Microbiol Immunol	Kramme et al.	Not POC. Laboratory-based
15	Detection of *Mycobacterium leprae* infection employing a combinatorial approach of anti-45 kDa and modified anti-PGL-I antibody detection assays.	2007	J Med Microbiol	Parkash et al.	Not POC. Laboratory-based / Publication type - research letter
16	Diagnostic value of in situ polymerase chain reaction in leprosy	2005	Indian Journal of Pediatrics	Dayal et al.	Not POC. Not accuracy study (Concordance between testes)
17	Early revelation of leprosy in china by sequential antibody analyses with LID-1 and PGL-I	2013	Journal of Tropical Medicine	Qiong-Hua et al.	Not accuracy study (Concordance between testes)
18	ELISA-based assay of immunoglobulin G antibodies against mammalian cell entry 1A (Mce1A) protein: a novel diagnostic approach for leprosy.	2017	Mem Inst Oswaldo Cruz	Lima et al.	Not accuracy study (Concordance between testes)
19	Estudo da sensibilidade e especificidade do teste Elisa anti PGL-1 no Estado de São Paulo TT - Sensibility study and specificity of ELISA test anti PGL-1 at São Paulo State	1997	Hansenol Int	Brasil et al.	Not POC. Laboratory-based
20	Evaluation of fluorescent staining for diagnosis of leprosy and its impact on grading of the disease: Comparison with conventional staining	2016	Journal of Clinical and Diagnostic Research	Adiga et al.	Not POC. Not accuracy study (Concordance between testes)

21	Evaluation of gelatin particle agglutination assay for the detection of anti-PGLI antibodies. Comparison with ELISA method and applicability on a large scale study using blood collected on filter paper.	1991	Lepr Rev	Chanteau et al.	Not POC. Not accuracy study (Concordance between testes)
22	Evaluation of major membrane protein-I as a serodiagnostic tool of pauci-bacillary leprosy	2014	Diagnostic Microbiology and Infectious Disease	Tsukamoto et al.	Not POC. Not accuracy study (Concordance between testes) Kappa
23	Evaluation of major membrane protein-II as a tool for serodiagnosis of leprosy.	2007	FEMS Microbiol Lett	Maeda et al.	Not accuracy study (Concordance between testes) Kappa
24	Evaluation of MLPA test for the serodiagnosis of leprosy.	1992	Int J Lepr Other Mycobact Dis	Dhandayuthapani et al.	Not POC. Not accuracy study (Concordance between testes)
25	Evaluation of modified lepro-agglutination as screening test for leprosy.	1994	Indian journal of leprosy	Thawani et al.	Not POC. Not accuracy study (Concordance between testes)
26	Evaluation of *Mycobacterium leprae* particle agglutination test, using eluates of filter paper blood spots.	1992	Lepr Rev	Sekar and Anandan	Not POC. Not accuracy study (Concordance between testes)
27	Evaluation of Polymerase Chain Reaction (PCR) with Slit Skin Smear Examination (SSS) to Confirm Clinical Diagnosis of Leprosy in Eastern Nepal	2016	PLoS Neglected Tropical Diseases	Siwakoti et al.	Accuracy, not POC
28	Evaluation of qPCR-Based assays for leprosy diagnosis directly in clinical specimens	2011	PLoS Neglected Tropical Diseases	Martinez et al.	Accuracy, not POC
29	FTA card utility for PCR detection of *Mycobacterium leprae*	2011	Japanese Journal of Infectious Diseases	Aye et al.	Not POC. Laboratory-based
30	Identifying Leprosy and Those at Risk of Developing Leprosy by Detection of Antibodies against LID-1 and LID-NDO	2016	PLoS Neglected Tropical Diseases	Amorim et al.	Not POC. Laboratory-based
31	Immunoglobulin class specific antibodies to M. leprae in leprosy patients, including the indeterminate group and healthy contacts as a step in the development of methods for sero-diagnosis of leprosy.	1982	Clin Exp Immunol	Melsom et al.	Not accuracy study
32	Leprosy reactions: The predictive value of *Mycobacterium leprae*-specific serology evaluated in a Brazilian cohort of leprosy patients (U-MDT/CT-BR)	2017	PLoS Neglected Tropical Diseases	Hungria et al.	Different population (leprosy reactions)
33	Leprosy serology (ML Flow test) in borderline leprosy patients classified as paucibacillary by counting cutaneous lesions: A useful tool	2008	Revista da Sociedade Brasileira de Medicina Tropical	Barreto et al.	Validation/Accuracy. Only for sensitivity
34	Low predictive value of PGL-I serology for the early diagnosis of leprosy in family contacts: Results of a 10-year prospective field study in French polynesia	1993	International Journal of Leprosy	Chanteau et al.	Not POC. Laboratory-based
35	Microtiter particle agglutination test for diagnosis of leprosy.	1992	Int J Lepr Other Mycobact Dis	Dyachina et al.	Not POC. Laboratory-based
36	Multiplex PCR technique could be an alternative approach for early detection of leprosy among close contacts - a pilot study from India	2009	BMC Infectious Diseases	Banerjee et al.	Not POC. Laboratory-based
37	Nasal PCR assay for the detection of *Mycobacterium leprae* pra gene to study subclinical infection in a community.	2017	Microb Pathog	Arunagiri et al.	Not POC. Laboratory-based
38	Novel gelatin particle agglutination test for serodiagnosis of leprosy in the field.	1990	J Clin Microbiol	Izumi et al.	Not accuracy study (Concordance between testes)
39	Performance of recombinant ESAT-6 antigen (ML0049) for detection of leprosy patients.	2007	Letters in Applied Microbiology	Parkash et al.	Not POC. Not accuracy study (Concordance between testes)
40	Rapid identification of *Mycobacterium leprae* by polymerase chain reaction-restriction fragment length polymorphism analysis of the heat shock protein 65 gene from skin specimens	2015	Chinese Medical Journal	Zhao et al.	Not accuracy study
41	Reverse transcription-PCR detection of *Mycobacterium leprae* in clinical specimens	1998	J Clin Microbiol	Kurabachew et al.	Not POC. Laboratory-based

42	Semi-quantitative detection of *Mycobacterium leprae* antigens in skin scrapings: suitability as a laboratory aid for field diagnosis of leprosy	2007	Transactions of the Royal Society of Tropical Medicine and Hygiene	Chaturvedi et al.	Not POC. Laboratory-based
43	Serological diagnosis of leprosy in patients in vietnam by enzyme-linked immunosorbent assay with *Mycobacterium leprae*-derived major membrane protein II.	2008	Clin Vaccine Immunol	Kai et al.	Not POC. Laboratory-based
44	Serological heterogeneity against various *Mycobacterium leprae* antigens and its use in serodiagnosis of leprosy patients [5]	2007	Journal of Medical Microbiology	Parkash et al.	Not POC. Not accuracy study (Concordance between testes)
45	Serological tests in leprosy. The sensitivity, specificity and predictive value of ELISA tests based on phenolic glycolipid antigens, and the implications for their use in epidemiological studies.	1988	Epidemiol Infect	Burgess et al.	Not POC. Laboratory-based
46	Serum IgA1 and IgM antibodies against *Mycobacterium leprae*-derived phenolic glycolipid-I: a comparative study in leprosy patients and their contacts.	1991	International journal of leprosy and other mycobacterial diseases	Chujor et al.	Not POC. Laboratory-based
47	Specific serological diagnosis of leprosy with a recombinant *Mycobacterium leprae* protein purified from a rapidly growing mycobacterial host.	1998	J Clin Microbiol	Triccas et al.	Not POC. Laboratory-based
48	The additional benefit of the ML Flow test to classify leprosy patients.	2009	Acta Tropica	Bührer-Sékula et al.	Different clinical outcome: Classification of leprosy patients
49	The ML flow test as a point of care test for leprosy control programmes: potential effects on classification of leprosy patients.	2007	Lepr Rev	Bührer-Sékula et al.	Different clinical outcome: Classification of leprosy patients
50	The result patterns of ML Flow and ELISA (PGL-I) serologic tests in leprosy-endemic and non-endemic areas.	2008	Revista da Sociedade Brasileira de Medicina Tropical	da Silva et al.	Not accuracy study (Concordance between testes) Kappa
51	The use of whole blood in a dipstick assay for detection of antibodies to *Mycobacterium leprae*: a field evaluation.	1998	FEMS Immunol Med Microbiol	Buhrer-Sekula et al.	Not accuracy study (Concordance between testes)
52	UltramicroELISA para la detección de anticuerpos IgM al *Mycobacterium leprae* utilizando muestras de sangre seca. TT - [Ultramicro ELISA to the detection of IgM antibodies in *Mycobacterium leprae* using dry blood samples].	1994	Rev Inst Med Trop Sao Paulo	Torrella et al.	Not POC test. Not accuracy study
53	Use of PCR-mediated amplification of *Mycobacterium leprae* DNA in different tupes of clinical samples for the diagnosis of leprosy	1993	J Med Microbiol	Santos et al.	Not POC. Laboratory-based
54	Use of the ML-Flow test as a tool in classifying and treating leprosy	2011	An Bras Dermatol	Contin et al.	Not accuracy study (Concordance between testes) Kappa
55	Utility of immunoglobulin isotypes against LID-1 and NDO-LID for, particularly IgG1, confirming the diagnosis of multibacillary leprosy.	2018	Mem Inst Oswaldo Cruz	Marçal et al.	Not POC. Laboratory-based
56	Utility of recombinant proteins LID-1 and PADL in screening for *Mycobacterium leprae* infection and leprosy	2014	Transactions of The Royal Society of Tropical Medicine and Hygiene	de Souza et al.	Not POC. Laboratory-based
57	Utility of serodiagnostic tests for leprosy: A study in an endemic population in South India	2004	Leprosy Review	Sinha et al.	Not POC. Laboratory-based
58	Comparison of synthetic antigens for detecting antibodies to phenolic glycolipid I in patients with leprosy and their household contacts	1988	J Infect Dis	Chanteau et al.	Not POC. Laboratory-based
59	Active search for leprosy cases in Midwestern Brazil: a serological evaluation of asymptomatic household contacts before and after prophylaxis with Bacillus Calmette-Guérin	2013	Rev. Inst. Med. Trop. Sao Paulo	Limeira et al.	Not POC. Laboratory-based
60	Development of a quantitative rapid diagnostic test for multibacillary leprosy using smart phone technology	2013	BMC Infectious Diseases	Cardoso et al.	Study Phase II – Development of test
61	Early detection of M. leprae by qPCR in untreated patients and their contacts: results for nasal swab and palate mucosa scraping.	2018	European journal of clinical microbiology & infectious diseases	Carvalho et al.	Not POC. Laboratory-based
62	Predictive value of gelatin particle agglutination test (GPAT) in leprosy detection	2018	Indian J. Lepr.	Khang et al.	Not POC. Laboratory-based

63	Application of new host biomarker profiles in quantitative point-of-care tests facilitates leprosy diagnosis in the field.	2019	EBioMedicine	van Hooij et al.	Study Phase II – Development of test
64	A novel integrated molecular and serological analysis method to predict new cases of leprosy amongst household contacts.	2019	PLoS neglected tropical diseases	Gama et al.	Not POC. Laboratory-based
65	Immunoglobulin AMG anti natural disaccharide octyl - Leprosy IDRI diagnostic (NDO-LID) serologic test for leprosy diagnosis: A pilot study	2019	Dermatol. Rep.	Rumondor et al.	Not POC. Laboratory-based
66	Quantitative polymerase chain reaction in paucibacillary leprosy diagnosis: A follow-up study.	2019	PLoS Negl Trop Dis	Barbieri et al.	Not POC. Laboratory-based
67	The use of synthetic glycoconjugates as components of the immunochromatographic test for rapid serological diagnosis of leprosy	2020	Klinicheskaia laboratornaia diagnostika	Korolyova-Ushakova et al.	Not POC. Laboratory-based
68	Pilot study to assess the accuracy and precision of phenolic glycolipid-i of *Mycobacterium leprae* test kit in diagnosing new leprosy cases among patients at the jose r. reyes memorial medical center	2020	J. Dermatol. Nurses' Assoc.	Montenegro et al.	Not POC. Laboratory-based
69	Single-nucleotide polymorphisms in genes predisposing to leprosy in leprosy household contacts in Zhejiang Province, China	2020	PharmacoEcon. Personalized Med.	Shen et al.	Not POC. Laboratory-based
70	Development of a novel loop-mediated isothermal amplification assay for rapid detection of *Mycobacterium leprae* in clinical samples.	2021	Indian J Dermatol Venereol Leprol	Joshi et al.	Not POC. Laboratory-based

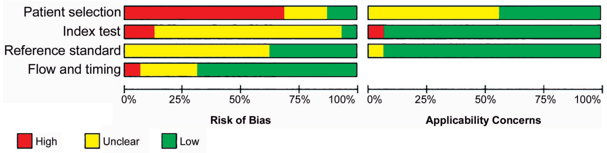

Fig. 1: risk of bias and applicability concerns graph: review authors' judgements about each domain presented as percentages across included studies.

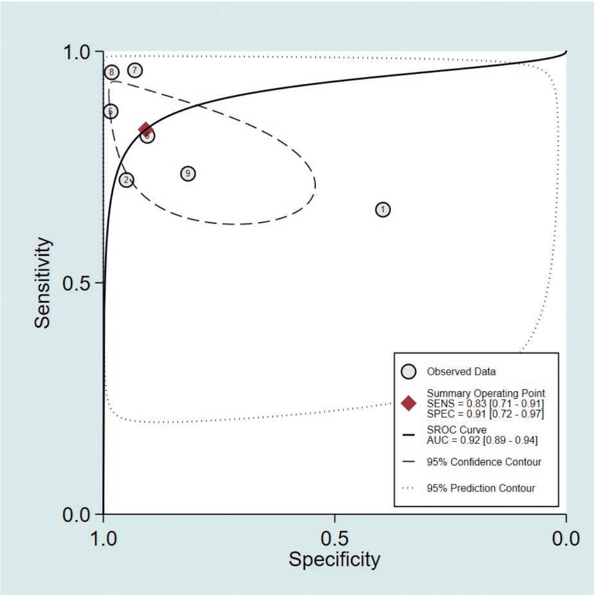

Fig. 2: SROC curve of NDO-LID tests for multibacillary cases. SROC curve with the pooled estimates of sensitivity, specificity and area under the curve. AUC: area under the curve; SENS: sensitivity; SPEC: specificity.

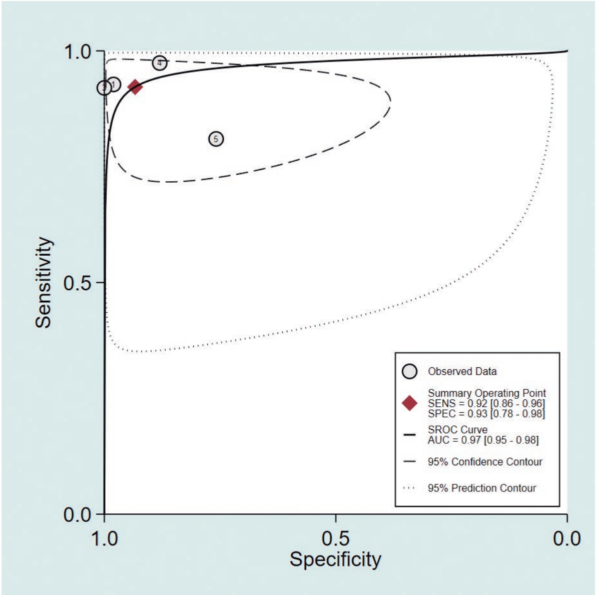

Fig. 3: SROC curve of PGL-I tests for multibacillary cases. SROC curve with the pooled estimates of sensitivity, specificity and area under the curve. AUC: area under the curve; SENS: sensitivity; SPEC: specificity.

TABLE III

Summary of tests accuracy parameters by multibacillary and paucibacillary of 16 included studies

					Overall					MB			PB			
ID	Author	Year	Cohort	Index test	TP	FN	FP	TN	*n*	TP	FN	*n*	TP	FN	*n*	% MB
a	Leturiondo AL[71]	2019	Brazil	NDO–LID conjugate	106	65	97	433	701	89	32	121	17	33	50	70.76%
b	Leturiondo AL[71]	2019	Brazil	Phenolic glycolipid-I (PGL-I)	114	57	128	402	701	98	23	121	16	34	50	70.76%
c	Góis R[72]	2018	Brazil	NDO–LID conjugate	66	4	1	54	125	21	1	22	45	3	48	31.43%
d	van Hooij A[73]	2018	Brazil, China, Ethiopia	PGL-I + (IP-10, CCL4 and CRP)	126	23	81	237	467	86	8	94	40	15	55	63.00%
e	Frade MAC[74]	2017	Brazil	NDO–LID conjugate	27	16	148	97	288	25	13	38	2	3	5	88.37%
f	van Hooij A[75]	2017	Philippines, Bangladesh	NDO–LID conjugate	158	116	8	152	434	148	57	205	10	59	69	74.82%
g	Duthie M[76]	2016	Philippines	NDO–LID conjugate	63	3	27	9	102	NA	NA	NA	NA	NA	NA	NA
h	Duthie M[76]	2016	Philippines	NDO–LID conjugate	51	15	0	36	102	NA	NA	NA	NA	NA	NA	NA
i	Duthie M[77]	2014	Philippines	NDO–LID conjugate	201	69	1	62	333	181	27	208	20	42	62	77.04%
j	Duthie M[77]	2014	Philippines	NDO–LID conjugate	174	96	6	57	333	170	38	208	4	58	62	77.04%
k	Duthie M[78]	2014	Colombia, Philippines	NDO–LID conjugate	169	13	8	109	299	140	6	146	37	13	50	74.49%
l	Stefani M[79]	2012	Brazil, Nepal	Phenolic glycolipid-I (PGL-I)	95	43	2	99	239	64	5	69	31	38	69	50.00%
m	Stefani M[79]	2012	Brazil, Nepal	Phenolic glycolipid-I (PGL-I)	87	52	NA	NA	139	63	6	69	24	46	70	49.64%
n	Parkash O[80]	2008	India	Phenolic glycolipid-I (PGL-I)	62	85	0	25	172	23	2	25	39	83	122	17.01%
o	Bührer-Sékula S[81]	2003	Brazil, Indonesia, Philippines, Ghana	Phenolic glycolipid-I (PGL-I)	145	54	28	206	433	111	3	114	34	51	85	57.29%
p	Roche P[82]	1999	Nepal	35-kD test card	59	28	1	9	97	38	7	45	21	15	36	55.56%

TP: true positive; FN: false negative; FP: false positive; TN: true negative; MB: multibacillary; PB: paucibacillary; Prev: prevalence in the studied sample; NA: not available.

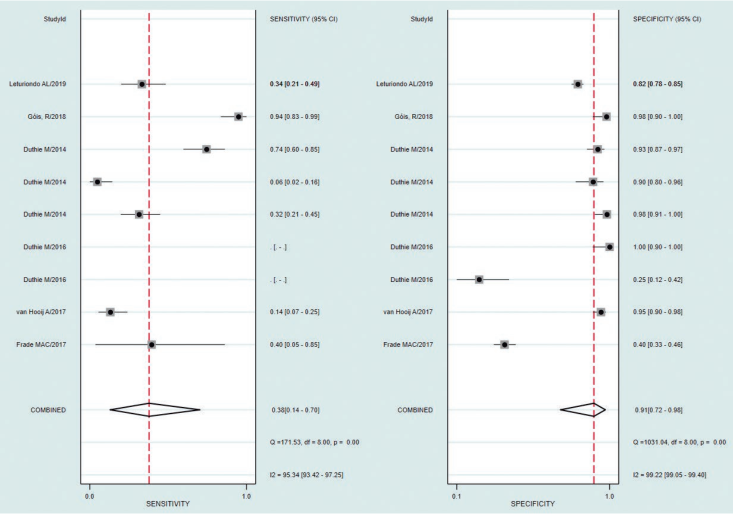

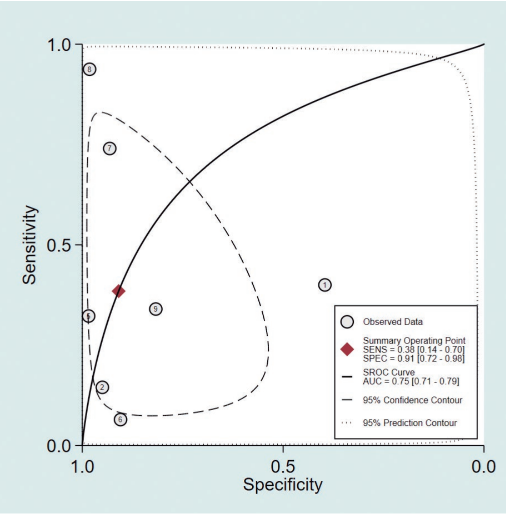

Figs 4-5: accuracy estimates of NDO-LID conjugate tests for paucibacillary cases. Forest plot showing sensitivity and specificity of NDO-LID tests for PB cases. SROC curve with the pooled estimates of sensitivity, specificity and area under the curve. AUC: area under the curve; SENS: sensitivity; SPEC: specificity.

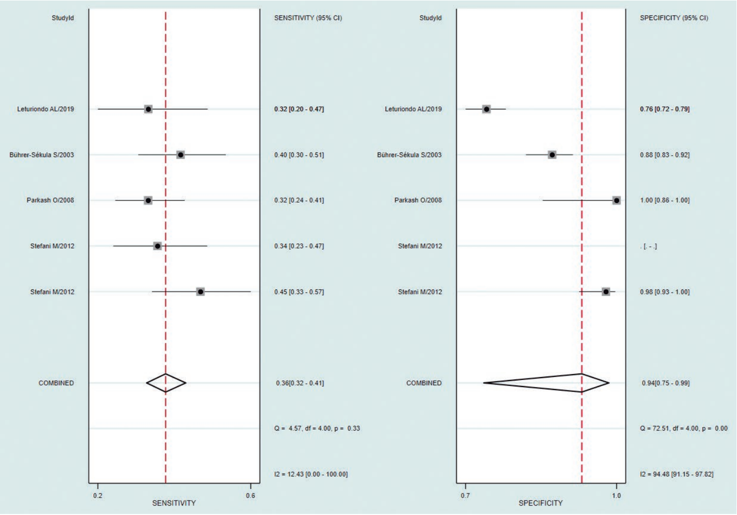

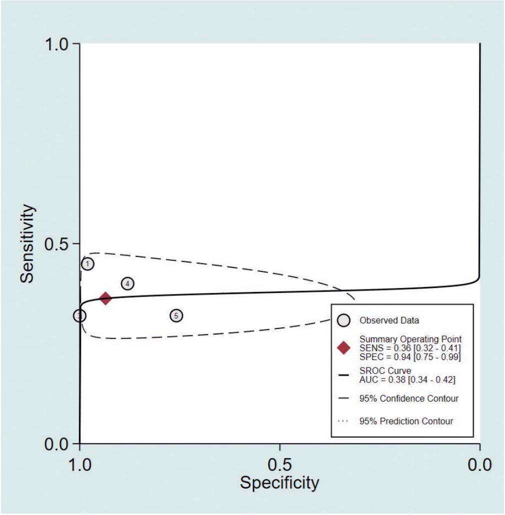

Figs 6-7: accuracy estimates of Phenolic glycolipid-I (PGL-I) conjugate tests for paucibacillary cases. Forest plot showing sensitivity and specificity of PGL-I tests for PB cases. SROC curve with the pooled estimates of sensitivity, specificity and area under the curve. AUC: area under the curve; SENS: sensitivity; SPEC: specificity.

REFERENCES

1. Ji BH, Tang QK, Li YL, Chen JK, Zhang JL, Dong LW, et al. The sensitivity and specificity of fluorescent leprosy antibody absorption (FLA-ABS) test for detecting subclinical infection with *Mycobacterium leprae*. Lepr Rev [Internet]. 1984;55(4):327–35. Available from: http://pesquisa.bvsalud.org/portal/resource/pt/mdl-6396473

2. Chanteau S, Cartel JL, Spiegel A, Plichart R, Roux J. [The detection of IgM antibodies to phenolglycolipid I for serodiagnosis of Hansen's disease and monitoring the contact population in Polynesia. Five year evaluation]. Bull Soc Pathol Exot. 1990;83(5):649–57.

3. Parkash O, Chaturvedi V, Girdhar BK, Sengupta U. A study on performance of two serological assays for diagnosis of leprosy patients . Lepr Rev . 1995 Mar;66(0305–7518):26–30.

4. Parkash O. A study on the reproducibility of two serological assays for detection of *Mycobacterium leprae* infection . IntJLeprOther MycobactDis [Internet]. 2001;69(0148-916X):46–8. Available from: http://www.embase.com/search/results?subaction=viewrecord&from=export&id=L32667339

5. Penna MLF, Penna GO, Iglesias PC, Natal S, Rodrigues LC. Anti-PGL-1 Positivity as a Risk Marker for the Development of Leprosy among Contacts of Leprosy Cases: Systematic Review and Meta-analysis. PLoS Negl Trop Dis [Internet]. 2016;10(5):1–11. Available from: http://www.embase.com/search/results?subaction=viewrecord&from=export&id=L610558098

6. Freitas AA, Hungria EM, Costa MBMB, Sousa ALOMLOM, Castilho MLO, Gonçalves HS, et al. Application of *Mycobacterium leprae*-specific cellular and serological tests for the differential diagnosis of leprosy from confounding dermatoses. Diagn Microbiol Infect Dis [Internet]. 2016 Oct;86(2):163–8. Available from: http://www.embase.com/search/results?subaction=viewrecord&from=export&id=L612461057

7. Chaturvedi V, Sinha S, Girdhar BK, Katoch K, Bhatia AS, Sengupta U. Association of mycobacterial-specific and *Mycobacterium leprae* specific antibody levels with clinical activity in tuberculoid leprosy: a comparative study of three serological enzyme-immunoassays. Lepr Rev. 1991 Jun;62(2):122–33.

8. Escobar-Gutierrez A, Amezcua ME, Pasten S, Pallares F, Cazares JV, Pulido RM, et al. Comparative assessment of the leprosy antibody absorption test, *Mycobacterium leprae* extract enzyme-linked immunosorbent assay, and gelatin particle agglutination test for serodiagnosis of lepromatous leprosy. J Clin Microbiol [Internet]. 1993;31(5):1329–33. Available from: http://pesquisa.bvsalud.org/portal/resource/pt/han-14235

9. Elston DM, Liranzo MO, Scollard DM. Comparing the sensitivity of auramine-rhodamine fluorescence to polymerase chain reaction in the detection of *Mycobacterium leprae* in Fite-negative tissue sections. J Am Acad Dermatol [Internet]. 2017;76(5):992–3. Available from: http://dx.doi.org/10.1016/j.jaad.2016.11.045

10. Maeda SM, Rotta O, Michalany NS, Camargo ZP, Sunderkötter C, Tomimori-Yamashita J, et al. Comparison between anti-PGL-I serology and Mitsuda reaction: clinical reading, microscopic findings and immunohistochemical analysis. Lepr Rev. 2003 Sep;74(3):263–74.

11. Cruz AF da, Furini RB, Roselino AMF. Comparison between microsatellites and Ml MntH gene as targets to identify *Mycobacterium leprae* by PCR in leprosy. An Bras Dermatol [Internet]. 2011;86(4):651–6. Available from: http://www.ncbi.nlm.nih.gov/pubmed/21987128

12. Lobato J, Costa MP, Reis EDM, Gonçalves MA, Spencer JS, Brennan PJ, et al. Comparison of three immunological tests for leprosy diagnosis and detection of subclinical infection. Lepr Rev [Internet]. 2011 Dec;82(4):389–401. Available from: http://www.embase.com/search/results?subaction=viewrecord&from=export&id=L364099244

13. Kang TJ, Kim SK, Lee SB, Chae GT, Kim JP. Comparison of two different PCR amplification products (the 18-kDa protein gene vs. RLEP repetitive sequence) in the diagnosis of *Mycobacterium leprae*. Clin Exp Dermatol [Internet]. 2003;28(4):420–4. Available from: http://www.embase.com/search/results?subaction=viewrecord&from=export&id=L36801849

14. Kramme S, Bretzel G, Panning M, Kawuma J, Drosten C. Detection and quantification of *Mycobacterium leprae* in tissue samples by real-time PCR. Med Microbiol Immunol [Internet]. 2004;193(4):189–93. Available from: http://pesquisa.bvsalud.org/portal/resource/pt/han-23200

15. Parkash O, Kumar A, Pandey R, Franken KLMCMC, Ottenhoff THMM. Detection of *Mycobacterium leprae* infection employing a combinatorial approach of anti-45 kDa and modified anti-PGL-I antibody detection assays [1]. J Med Microbiol [Internet]. 2007;56(8):1129–30. Available from: http://pesquisa.bvsalud.org/portal/resource/pt/mdl-17644727

16. Dayal R, Singh SP, Mathur PP, Katoch VM, Katoch K, Natrajan M, et al. Diagnostic value of in situ polymerase chain reaction in leprosy. Indian J Pediatr [Internet]. 2005;72(12):1043–6. Available from: http://www.embase.com/search/results?subaction=viewrecord&from=export&id=L43075132

17. Qiong-Hua P, Zhong-Yi Z, Jun Y, Yan W, Lian-Chao Y, Huan-Ying L, et al. Early revelation of leprosy in china by sequential antibody analyses with LID-1 and PGL-I. J Trop Med [Internet]. 2013;2013. Available from: http://www.embase.com/search/results?subaction=viewrecord&from=export&id=L368457723

18. Lima FR, Takenami I, Cavalcanti M Al, Riley LW, Arruda S. ELISA-based assay of immunoglobulin G antibodies against mammalian cell entry 1A (Mce1A) protein: a novel diagnostic approach for leprosy. Mem Inst Oswaldo Cruz. 2017 Dec;112(12):844–9.

19. Brasil MTLRF, Oliveira LR, de Mello CS, Nakamura PM, Manini MP, Steiner D, et al. Estudo da sensibilidade e especificidade do teste Elisa anti PGL-1 no Estado de São Paulo TT - Sensibility study and specificity of ELISA test anti PGL-1 at São Paulo State. Hansen Int [Internet]. 1997;22(2):35–43. Available from: http://hansen.bvs.ilsl.br/textoc/hansenint/v21aov29/1997/PDF/v22n2/v22n2a05.pdf

20. Adiga DSA, Hippargi SB, Rao G, Saha D, Yelikar BR, Karigoudar M, et al. Evaluation of Fluorescent Staining for Diagnosis of Leprosy and its Impact on Grading of the Disease: Comparison with Conventional Staining. J Clin Diagn Res [Internet]. 2016 Oct;10(10):EC23–6. Available from: http://www.embase.com/search/results?subaction=viewrecord&from=export&id=L612772538

21. Chanteau S, Cartel JL, Boutin JP, Roux J. Evaluation of gelatin particle agglutination assay for the detection of anti-PGLI antibodies. Comparison with ELISA method and applicability on a large scale study using blood collected on filter paper . Lepr Rev [Internet]. 1991 Sep;62(0305–7518):255–61. Available from: http://pesquisa.bvsalud.org/portal/resource/pt/mdl-1795583

22. Tsukamoto Y, Maeda Y, Makino M, et al. Evaluation of major membrane protein-I as a serodiagnostic tool of pauci-bacillary leprosy. Diagn Microbiol Infect Dis [Internet]. 2014 Sep;80(1):62–5. Available from: http://www.embase.com/search/results?subaction=viewrecord&from=export&id=L53245595

23. Maeda Y, Mukai T, Kai M, Fukutomi Y, Nomaguchi H, Abe C, et al. Evaluation of major membrane protein-II as a tool for serodiagnosis of leprosy. FEMS Microbiol Lett. 2007 Jul;272(2):202–5.

24. Dhandayuthapani S, Anandan D, Bhatia VN. Evaluation of MLPA test for the serodiagnosis of leprosy. Int J Lepr Other Mycobact Dis [Internet]. 1992;60(1):84–7. Available from: http://pesquisa.bvsalud.org/portal/resource/pt/mdl-1602199

25. Thawani G, Mukherjee A, Bhatia VN. Evaluation of modified lepro-agglutination as screening test for leprosy. Indian J Lepr. 1994;66(3):315–20.

26. Sekar B, Anandan D. Evaluation of *Mycobacterium leprae* particle agglutination test, using eluates of filter paper blood spots. Lepr Rev [Internet]. 1992 Jun;63(2):117–24. Available from: http://pesquisa.bvsalud.org/portal/resource/pt/mdl-1640778

27. Siwakoti S, Rai K, Bhattarai NR, Agarwal S, Khanal B. Evaluation of Polymerase Chain Reaction (PCR) with Slit Skin Smear Examination (SSS) to Confirm Clinical Diagnosis of Leprosy in Eastern Nepal. PLoS Negl Trop Dis [Internet]. 2016;10(12):e0005220–e0005220. Available from: http://www.embase.com/search/results?subaction=viewrecord&from=export&id=L613987301

28. Martinez ANN, Ribeiro-Alves M, Sarno EN, Moraes MO. Evaluation of qPCR-Based assays for leprosy diagnosis directly in clinical specimens. PLoS Negl Trop Dis [Internet]. 2011 Oct;5(10):1–8. Available from: http://www.embase.com/search/results?subaction=viewrecord&from=export&id=L362834568

29. Aye KS, Matsuoka M, Kai M, Kyaw K, Shwe MM, et al. FTA card utility for PCR detection of *Mycobacterium leprae*. Jpn J Infect Dis [Internet]. 2011;64(3):246–8. Available from: http://www.embase.com/search/results?subaction=viewrecord&from=export&id=L361894984

30. Amorim FM, Nobre ML, Ferreira LC, Nascimento LS, Miranda AM, Monteiro GRG, et al. Identifying Leprosy and Those at Risk of Developing Leprosy by Detection of Antibodies against LID-1 and LID-NDO. PLoS Negl Trop Dis. 2016;10(9).

31. Melsom R, Harboe M, Myrvang B, Godal T, Belehu A. Immunoglobulin class specific antibodies to M. leprae in leprosy patients, including the indeterminate group and healthy contacts as a step in the development of methods for sero-diagnosis of leprosy. Clin Exp Immunol. 1982 Feb;47(2):225–33.

32. Hungria EM, Bührer-Sékula S, de Oliveira RM, Aderaldo LC, Pontes ADA, Cruz R, et al. Leprosy reactions: The predictive value of *Mycobacterium leprae*-specific serology evaluated in a Brazilian cohort of leprosy patients (U-MDT/CT-BR). PLoS Negl Trop Dis [Internet]. 2017;11(2):e0005396–e0005396. Available from: http://dx.doi.org/10.1371/journal.pntd.0005396

33. Barreto JA, Nogueira MES, Diorio SM, Bührer-Sékula S. Leprosy serology (ML Flow test) in borderline leprosy patients classified as paucibacillary by counting cutaneous lesions: A useful tool. Rev Soc Bras Med Trop [Internet]. 2008;41(SUPPL. 2):45–7. Available from: http://www.embase.com/search/results?subaction=viewrecord&from=export&id=L355314632

34. Chanteau S, Glaziou P, Plichart C, Luquiaud P, Plichart R, Faucher JF, et al. Low predictive value of PGL-I serology for the early diagnosis of leprosy in family contacts: Results of a 10-year prospective field study in French polynesia. Int J Lepr [Internet]. 1993;61(4):533–41. Available from: http://www.embase.com/search/results?subaction=viewrecord&from=export&id=L24129408

35. Dyachina MN, Lukin Y V, Zubov VP, Bovin N V. Microtiter particle agglutination test for diagnosis of leprosy. Int J Lepr Other Mycobact Dis [Internet]. 1992;60(4):575–9. Available from: http://pesquisa.bvsalud.org/portal/resource/pt/mdl-1299713

36. Banerjee S, Sarkar K, Gupta S, Mahapatra PS, Gupta S, Guha S, et al. Multiplex PCR technique could be an alternative approach for early detection of leprosy among close contacts - a pilot study from India. BMC Infect Dis [Internet]. 2009;10:252. Available from: http://www.embase.com/search/results?subaction=viewrecord&from=export&id=L51044529

37. Arunagiri K, Sangeetha G, Sugashini PK, Balaraman S, Showkath Ali MK. Nasal PCR assay for the detection of *Mycobacterium leprae* pra gene to study subclinical infection in a community. Microb Pathog [Internet]. 2017 Mar;104:336–9. Available from: http://dx.doi.org/10.1016/j.micpath.2017.01.046

38. Izumi S, Fujiwara T, Ikeda M, Nishimura Y, Sugiyama K, Kawatsu K. Novel gelatin particle agglutination test for serodiagnosis of leprosy in the field. J Clin Microbiol [Internet]. 1990 Mar;28(3):525–9. Available from: http://www.ncbi.nlm.nih.gov/pmc/articles/PMC269656/?tool=pubmed

39. Parkash O, Kumar A, Pandey R, Girdhar BK, Franken KLMC, Ottenhoff THM, et al. Serological heterogeneity against various *Mycobacterium leprae* antigens and its use in serodiagnosis of leprosy patients [5]. J Med Microbiol [Internet]. 2007;56(9):1259–61. Available from: http://www.embase.com/search/results?subaction=viewrecord&from=export&id=L47477205

40. Zhao Z, Liu XW, Jia J, Cai L, Zhang JZ. Rapid Identification of *Mycobacterium Leprae* by Polymerase Chain Reaction restriction Fragment Length Polymorphism Analysis of the Heat Shock Protein 65 Gene from Skin Specimens. Chin Med J (Engl) [Internet]. 2015;128(21):2964–6. Available from: http://www.embase.com/search/results?subaction=viewrecord&from=export&id=L606630956

41. Kurabachew M, Wondimu A, Ryon JJ. Reverse transcription-PCR detection of *Mycobacterium leprae* in clinical specimens. J Clin Microbiol [Internet]. 1998;36(5):5. Available from: http://www.ncbi.nlm.nih.gov/pmc/articles/PMC104827/?tool=pubmed

42. Chaturvedi V, Girdhar BK, Sengupta U, Sinha S. Semi-quantitative detection of *Mycobacterium leprae* antigens in skin scrapings: suitability as a laboratory aid for field diagnosis of leprosy. Trans R Soc Trop Med Hyg [Internet]. 2007 Jul;101(7):699–706. Available from: http://www.embase.com/search/results?subaction=viewrecord&from=export&id=L46706156

43. Kai M, Nguyen Phuc NH, Hoang Thi TH, Nguyen AH, Fukutomi Y, Maeda Y, et al. Serological diagnosis of leprosy in patients in vietnam by enzyme-linked immunosorbent assay with *Mycobacterium leprae*-derived major membrane protein II. Clin Vaccine Immunol [Internet]. 2008 Dec;15(12):1755–9. Available from: http://www.ncbi.nlm.nih.gov/pmc/articles/PMC2593173/?tool=pubmed

44. Parkash O, Kumar A, Pandey R, Franken KLMCMC, Ottenhoff THMM. Detection of *Mycobacterium leprae* infection employing a combinatorial approach of anti-45 kDa and modified anti-PGL-I antibody detection assays [1]. J Med Microbiol [Internet]. 2007;56(8):1129–30. Available from: http://pesquisa.bvsalud.org/portal/resource/pt/mdl-17644727

45. Burgess PJ, Fine PEM, Ponnighaus JM, Draper C. Serological tests in leprosy. The sensitivity, specificity and predictive value of ELISA tests based on phenolic glycolipid antigens, and the implications for their use in epidemiological studies. Epidemiol Infect [Internet]. 1988;101(1):159–71. Available from: http://www.ncbi.nlm.nih.gov/pmc/articles/PMC2249338/?tool=pubmed

46. Chujor CS, Bernheimer H, Levis WR, Schwerer B. Serum IgA1 and IgM antibodies against *Mycobacterium leprae*-derived phenolic glycolipid-I: a comparative study in leprosy patients and their contacts. Int J Lepr Other Mycobact Dis. 1991 Sep;59(3):441–9.

47. Triccas JA, Roche PW, Britton WJ. Specific serological diagnosis of leprosy with a recombinant *Mycobacterium leprae* protein purified from a rapidly growing mycobacterial host. J Clin Microbiol [Internet]. 1998 Aug;36(8):2363–5. Available from: http://www.ncbi.nlm.nih.gov/pmc/articles/PMC105051/?tool=pubmed

48. Bührer-Sékula S, Illarramendi X, Teles RB, Penna MLF, Nery JAC, Sales AM, et al. The additional benefit of the ML Flow test to classify leprosy patients. Acta Trop [Internet]. 2009;111(2):172–6. Available from: http://dx.doi.org/10.1016/j.actatropica.2009.04.009

49. Bührer-Sékula S, Visschedijk J, Grossi MAF, Dhakal KP, Namadi AU, Klatser PR, et al. The ML flow test as a point of care test for leprosy control programmes: potential effects on classification of leprosy patients. Lepr Rev. 2007;78(June 2014):70–9.

50. Silva RC da, Lyon SHSSH, Araos R, Lyon ACC, Grossi MADF, Lyon SHSSH, et al. The result patterns of ML Flow and ELISA (PGL-I) serologic tests in leprosy-endemic and non-endemic areas. Rev Soc Bras Med Trop. 2008;41(Suplemento II):19–22.

51. Buhrer-Sekula S, Cunha MG da GS, Ferreira WA, Klatser PR, Bührer-Sekula S, Cunha MG da GS, et al. The use of whole blood in a dipstick assay for detection of antibodies to *Mycobacterium leprae*: a field evaluation. FEMS Immunol Med Microbiol [Internet]. 1998 Jul;21(3):197–201. Available from: http://pesquisa.bvsalud.org/portal/resource/pt/mdl-9718209

52. Torrella A, Solis RL, Rodriguez N, Medina Y, Pita M, Perez I, et al. UltramicroELISA para la detección de anticuerpos IgM al *Mycobacterium leprae* utilizando muestras de sangre seca. Rev Inst Med Trop Sao Paulo. 1994;36(2):131–8.

53. Santos AR, Demiranda AB, Sarno EN, Suffys PN, Degrave WM. Use of Pcr-Mediated Amplification of Mycobacterium-Leprae DNA in Different Types of Clinical-Samples for the Diagnosis of Leprosy. J Med Microbiol. 1993;39(4):298–304.

54. Contin LA, Alves CJM, Fogagnolo L, Nassif PW, Barreto JA, Lauris JRP, et al. Use of the ML-Flow test as a tool in classifying and treating leprosy. An Bras Dermatol [Internet]. 2011;86(1):91–5. Available from: http://www.scielo.br/scielo.php?script=sci_arttext&nrm=iso&lng=pt&tlng=pt&pid=S0365-05962011000100012

55. Marçal PH, Fraga LA, Ottenhoff T, Geluk A, Duthie, M, Teixeira HC. Immune response to recombinant proteins of *Mycobacterium leprae* potential application for leprosy diagnosis. Am J Trop Med Hyg [Internet]. 2017;97(5):236. Available from: http://www.embase.com/search/results?subaction=viewrecord&from=export&id=L620729647

56. de Souza MM, Netto EM, Nakatani M, Duthie MS. Utility of recombinant proteins LID-1 and PADL in screening for *Mycobacterium leprae* infection and leprosy. Trans R Soc Trop Med Hyg [Internet]. 2014 Aug;108(8):495–501. Available from: https://academic.oup.com/trstmh/article-lookup/doi/10.1093/trstmh/tru093

57. Sinha S, Kannan S, Nagaraju B, Sengupta U, Gupte M D. Utility of serodiagnostic tests for leprosy: A study in an endemic population in South India. Lepr Rev [Internet]. 2004 Sep;75(0305–7518):266–73. Available from: http://www.embase.com/search/results?subaction=viewrecord&from=export&id=L39362687

58. Chanteau S, Cartel JL, Roux J, Plichart R, Bach MA. Comparison of synthetic antigens for detecting antibodies to phenolic glycolipid I in patients with leprosy and their household contacts. J Infect Dis [Internet]. 1988 Apr;157(4):770–6. Available from: http://pesquisa.bvsalud.org/portal/resource/pt/mdl-3346568

59. Limeira OM, Gomes CM, Morais OO de, Cesetti MV, Alvarez RRA. Active search for leprosy cases in Midwestern Brazil: a serological evaluation of asymptomatic household contacts before and after prophylaxis with bacillus Calmette-Guérin. Rev Inst Med Trop Sao Paulo [Internet]. 2013;55(3):173–7. Available from: http://www.scielo.br/scielo.php?script=sci_arttext&pid=S0036-46652013000300173&lng=en&tlng=en

60. Paula Vaz Cardoso L, Dias RF, Freitas AA, Hungria EM, Oliveira RM, Collovati M, et al. Development of a quantitative rapid diagnostic test for multibacillary leprosy using smart phone technology. BMC Infect Dis [Internet]. 2013;13(1):497. Available from: http://www.embase.com/search/results?subaction=viewrecord&from=export&id=L52836454

61. Carvalho RS, Foschiani IM, Costa MRSN, Marta SN, da Cunha Lopes Virmond M. Early detection of M. leprae by qPCR in untreated patients and their contacts: results for nasal swab and palate mucosa scraping. Eur J Clin Microbiol Infect Dis. 2018;37(10):1863–7.

62. Khang TH, Thanh LT, Lanh PH. Predictive value of gelatin particle agglutination test (GPAT) in leprosy detection. Indian J Lepr. 2018;90(1):61–7.

63. van Hooij A, van den Eeden S, Richardus R, Tjon Kon Fat E, Wilson L, Franken KLMC, et al. Application of new host biomarker profiles in quantitative point-of-care tests facilitates leprosy diagnosis in the field. EBioMedicine. 2019;47:301–8.

64. Gama RS, de Souza MLM, Sarno EN, de Moraes MO, Gonçalves A, Stefani MMA, et al. A novel integrated molecular and serological analysis method to predict new cases of leprosy amongst household contacts. PLoS Negl Trop Dis. 2019;13(6):1–22.

65. Rumondor BB, Prakoeswa AC, Trianita MN, Iswahyudi, Herwanto N, Listiawan MY, et al. Immunoglobulin AMG anti natural disaccharide octyl - Leprosy IDRI diagnostic (NDO-LID) serologic test for leprosy diagnosis: A pilot study. Dermatology Reports. 2019;11(S1):31–4.

66. Barbieri RR, Manta FSN, Moreira SJM, Sales AM, Nery JAC, Nascimento LPR, et al. Quantitative polymerase chain reaction in paucibacillary leprosy diagnosis: A follow-up study. PLoS Negl Trop Dis. 2019;13(3):1–12.

67. Korolyova-Ushakova AG, Baranova E V., Ignatov SG, Fedyukina GN, Solov'ev P V., Kolombet L V., et al. the Use of Synthetic Glycoconjugates As Components of the Immunochromatographic Test for Rapid Serological Diagnosis of Leprosy. Russ Clin Lab Diagnostics. 2020;65(5):289–93.

68. Montenegro MK, Venida-tablizo A, Abad-Venida ML. Pilot study to assess the accuracy and precision of phenolic glycolipid-i of *Mycobacterium leprae* test kit in diagnosing new leprosy cases among patients at the jose r. reyes memorial medical center. J. Dermatol. Nurses' Assoc. - Volume 12, Issue 2, pp.

69. Shen YL, Long SY, Kong WM, Wu LM, Fei LJ, Yao Q, et al. Single-nucleotide polymorphisms in genes predisposing to leprosy in leprosy household contacts in Zhejiang Province, China. Pharmgenomics Pers Med. 2020;13:767–73.

70. Joshi S, Sharma V, Ramesh V, Singh R, Salotra P. Development of a novel loop-mediated isothermal amplification assay for rapid detection of *Mycobacterium leprae* in clinical samples. Indian J Dermatol Venereol Leprol - Volume 0, Issue 0, pp. 1-7.

71. Leturiondo AL, Noronha AB, do Nascimento MOO, Ferreira C de O, Rodrigues F da C, Moraes MO, et al. Performance of serological tests PGL1 and NDO-LID in the diagnosis of leprosy in a reference Center in Brazil. BMC Infect Dis. 2019;19(1):1–6.

72. Góis RV, Travaim SF, Prata GMC, Degen AN, Pereira GCA, Wolf JM, et al. Avaliação do desempenho de um teste rápido imunocromatográfico no diagnóstico de hanseníase em uma região endêmica no norte do Brasil. Clin Biomed Res. 2018;38(4):348–55.

73. van Hooij A, Tjon Kon Fat EM, Batista da Silva M, Carvalho Bouth R, Cunha Messias AC, Gobbo AR, et al. Evaluation of Immunodiagnostic Tests for Leprosy in Brazil, China and Ethiopia. Sci Rep. 2018;8(1):1–9.

74. Frade MAC, de Paula NANA, Gomes CM, Vernal S, Bernardes Filho F, Lugão HB, et al. Unexpectedly high leprosy seroprevalence detected using a random surveillance strategy in midwestern Brazil: A comparison of ELISA and a rapid diagnostic test. Johnson C, editor. PLoS Negl Trop Dis [Internet]. 2017 Feb 23;11(2):e0005375–e0005375. Available from: http://dx.doi.org/10.1371/journal.pntd.0005375

75. van Hooij A, Tjon Kon Fat EM, van den Eeden SJF, Wilson L, Batista da Silva M, Salgado CG, et al. Field-friendly serological tests for determination of M. leprae-specific antibodies. Sci Rep. 2017 Aug;7(1):8868.

76. Duthie MS, Orcullo FM, Abbelana J, Maghanoy A, Balagon MF. Comparative evaluation of antibody detection tests to facilitate the diagnosis of multibacillary leprosy. Appl Microbiol Biotechnol [Internet]. 2016 Apr 28;100(7):3267–75. Available from: http://www.embase.com/search/results?subaction=viewrecord&from=export&id=L607996607

77. Duthie MS, Balagon MF, Maghanoy A, Orcullo FM, Cang M, Dias RF, et al. Rapid quantitative serological test for detection of infection with *Mycobacterium leprae*, the causative agent of leprosy. J Clin Microbiol [Internet]. 2014;52(2):613–9. Available from: http://www.embase.com/search/results?subaction=viewrecord&from=export&id=L372222888

78. Duthie M, Raychaudhuri R, Tutterrow Y L, Misquith A, Bowman J, Casey A, et al. A rapid ELISA for the diagnosis of MB leprosy based on complementary detection of antibodies against a novel protein-glycolipid conjugate. Diagn Microbiol Infect Dis [Internet]. 2014;79(2):233–9. Available from: http://dx.doi.org/10.1016/j.diagmicrobio.2014.02.006

79. Stefani MM de A, Grassi AB, Sampaio LH, Sousa ALOM de, Costa MB, Scheelbeek P, et al. Comparison of two rapid tests for anti-phenolic glycolipid-I serology in Brazil and Nepal. Mem Inst Oswaldo Cruz [Internet]. 2012;107(supl.1):124–31. Available from: http://www.scielo.br/scielo.php?script=sci_arttext&pid=S0074-02762012000900019

80. Parkash O, Kumar A, Pandey R, Nigam A, Girdhar BK. Performance of a lateral flow test for the detection of leprosy patients in India [2]. J Med Microbiol [Internet]. 2008;57(1):130–2. Available from: http://www.embase.com/search/results?subaction=viewrecord&from=export&id=L351120591

81. Bührer-Sékula S, Smits HL, Gussenhoven GC, van Leeuwen J, Amador S, Fujiwara T, et al. Simple and fast lateral flow test for classification of leprosy patients and identification of contacts with high risk of developing leprosy. J Clin Microbiol [Internet]. 2003;41(5):5. Available from: http://pesquisa.bvsalud.org/portal/resource/pt/han-20768

82. Roche PW, Failbus SS, Britton WJ, Cole R. Rapid method for diagnosis of leprosy by measurements of antibodies to the M. leprae 35-kDa protein: comparison with PGL-I antibodies detected by ELISA and "dipstick" methods. Int J Lepr Other Mycobact Dis [Internet]. 1999 Sep;67(3):279–86. Available from: http://pesquisa.bvsalud.org/portal/resource/pt/mdl-10575407.
